# Community engagement in kidney research: Guatemalan experience

**DOI:** 10.1186/s12882-022-02891-8

**Published:** 2022-08-12

**Authors:** Angie Aguilar-González, Randall Lou-Meda, André Chocó-Cedillos, Louise Moist

**Affiliations:** 1 Foundation for Children With Kidney Disease -FUNDANIER-, 6 avenida 9-18 zona 10, torre 1, Oficina 804, Edificio Sixtino 2, Guatemala, 01010 Guatemala; 2 Division of Nephrology, Department of Medicine, Schulich School of Medicine and Dentistry, Western University, London, Canada; 3 Kidney Clinical Research Unit, London Health Sciences Centre, London, Canada

**Keywords:** Community engagement, Guatemala, Community participation, Evaluation, Community program

## Abstract

**Background:**

Community engagement is essential for effective research when addressing issues important to both the community and researchers. Despite its effectiveness, there is limited published evidence concerning the evaluation of community engagement in research projects, especially in the area of nephrology.

**Methods:**

We developed a community engagement program in Guatemala to address the role of hydration in chronic kidney disease of unknown origin, using five key engagement principles: 1. Local relevance and determinants of health. 2. Acknowledgment of the community. 3. Dissemination of findings and knowledge gained to all partners. 4. Usage of community partners’ input. 5. Involvement of a cyclical and iterative process in the pursuit of goals. The effectiveness of community engagement was measured by a structured questionnaire on a 5-point likert scale. This measure determined how well and how often the research team adhered to the five engagement principles. We assessed internal consistency for each set of the engagement items through Omega coefficient.

**Results:**

Sixty-two community leaders completed the questionnaire. Seventy-five percent were female, with a mean age of 37 years. All 5 engagement principles scored highly on the 5-point likert scale. Every item set corresponding to an engagement principles evaluation had a Omega coefficient > 0.80, indicating a firm internal consistency for all question groups on both qualitative and quantitative scales.

**Conclusion:**

Engagement of the community in the kidney research provides sustainability of the efforts and facilitates the achievements of the goals. Community leaders and researchers became a team and develop a relationship in which commitment and empowerment facilitated the participation in all aspects of the research process. This initiative could be a useful tool for researchers, especially in low-middle income countries, to start research in a community, achieve objectives in a viable form, and open opportunities to further studies.

**Supplementary Information:**

The online version contains supplementary material available at 10.1186/s12882-022-02891-8.

## Background

Collaborative research, involving coordination between researchers, organizations and communities, provides opportunities for investigators to learn how approaches from complementary disciplines may be applied to existing problems, and lead to the development of innovative and simple solution [1–4]. Collaboration strengthens the establishment of effective communication, offers equal opportunities among the team members and consolidates their partnership [5–8]. Collaboration also increases the ethical conduct maintaining respect, honesty, integrity, justice, transparency, and confidentiality [2–4, 9, 10].

Community engagement has emerged as an evidence-based approach in conducting research with the community as a lead partnering to better respond to complex issues arising in the community [1, 4]. While developing an engagement program, the community members become a part of the research team, ensuring the focus remains on the important issues faced by the constituents [6–11]. The value of engaging helps to re-orient and to improve research enterprise goals, patient care, decision-making process, and health outcomes by increasing the relevance of the research to the community [3, 6, 11–13]. It also assists with the knowledge translation of the research outcomes as the stakeholders are already engaged and knowledgeable [5, 14–17].

Despite community engagement program effectiveness, there is limited evidence in the literature concerning the evaluation of community engagement in research projects [17] especially in the area of kidney health and disease. Global evidence repeatedly shows that there is an implementation gap between Chronic Kidney Disease (CKD) guidelines and clinical practice [7, 18–20]. This includes variable levels of recognition, difficulties in communication, poor patient awareness and uncertainty surrounding medication and referrals [7, 18–20]. One way to address such gaps and optimize delivery of patient-centered CKD care is to engage the community in kidney research [7, 18–20]. Redirecting the focus of research to community priorities may enhance the relevance and uptake of evidence into practice and provide solutions to improve public health, community well-being and to eliminate health disparities in CKD [7, 18–20].

An increase in CKD prevalence related to non-traditional risk factors, such as dehydration primarily affecting male agricultural workers has been reported in several tropical countries [21–29]. Despite several years of research and risk factors identification the etiology of this Chronic Kidney Disease of unknown origin (CKDu) remains elusive [7, 28]. Recurrent dehydration is believed to be a contributor to CKDu among people with prolonged exposure to heat in both outdoor and indoor environments [30]. However, there are many cases reported, including women and children who live in the same environment presenting with kidney diseases [31, 32]. Epidemiologic studies in Guatemala demonstrate a high prevalence of kidney diseases in municipalities along the pacific coast [33]. Seventy eight percent of patients come from 61 municipalities in this region. When compared with other rural areas in the country, the municipality of La Democracia in southern Guatemala has the highest prevalence of the kidney disease [33]. Studies in Guatemalan children with CKD found that in 43% of patients the cause of CKD was not identified [34]. Adequate hydration is essential for healthy kidneys. This community engagement initiative was developed as an essential step in a kidney research project designed to evaluate the intake of fluid in healthy Guatemalan children and adolescents, considering amounts, types of beverages, and time of consumption in an area of high prevalence of CKDu in Guatemala.

Engagement principles are facilitative to enhancing health promotion and research [35, 36]. These principles have considered: items before beginning engagement, what is necessary for engagement to occur and what to consider for engagement to be successful [37]. The community focuses on issues important to the community, knows what is going on with the project and their input helps to make decisions at multiple stages of the process [35–37]. Research projects become community priorities and the community is involved throughout the entire research process [5, 38, 39].

The objective of this study is to evaluate the community engagement initiative, on stakeholders and leaders as the community of interest, using a previously published structured questionnaire, to assess engagement effectiveness on a quantitative and qualitative scale.

## Methods

### Study design

We developed a community engagement program in 2018 in the southern region of Guatemala and evaluated its effectiveness based on five engagement principles previously established in the literature [33]. The five engagement principles include: (1) Local relevance and determinants of health; (2) Acknowledgment of the community; (3) Dissemination of findings and knowledge gained to all partners; (4) Usage of community partner input; (5) Involvement of a cyclical and iterative process in the pursuit of objectives.

### Population

The project took place in the Municipality of La Democracia, Escuintla, Guatemala, one of the municipalities with the highest prevalence of CKD [33]. The municipality is located 165 m above sea level in the center of the province of Escuintla [38]. La Democracia has a territorial extension of 320 Km^2^, and it is 92 km away from the Guatemala City [40]. The population in La Democracia was 29,604 in 2019, representing 4% of the total population of the Province of Escuintla [40, 41]. Sugar cane agribusiness is the main economic activity that sustains the livelihood of the community [40]. Free access to health services is provided in the Community Health Clinic, comprised of emergency service and four outpatient clinics with basic medical equipment. The rural branch clinic staffed by a nurse's aide is located in Parcelamiento El Pilar. At La Democracia there are 3.3 health professionals (general practitioners and nurses) for every 10,000 inhabitants [40].

### Development of a Community Engagement Program

**Fig. 1:**
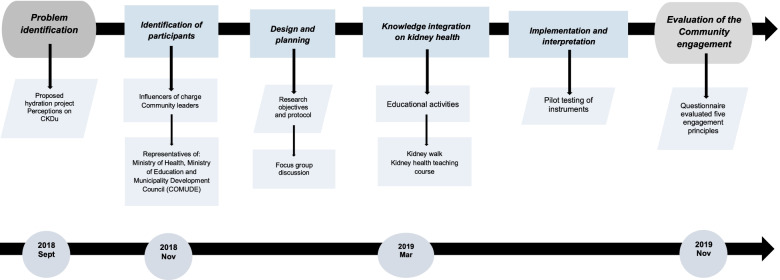
Development of a Community Engagement Program

Key elements included selection of stakeholders, problem identification, participant invitation, design and planning, knowledge gain, enrolling, implementation and interpretation are described on details below (Fig. 1).

#### Problem identification

The research team, composed of 2 pediatric nephrologists and a general doctor approached the only community health clinic in La Democracia. A medical director, medical assistant, and epidemiologist of the clinic expressed their opinion about perceptions on CKDu. During this approach, led by the research team, we understood how community was structured and met in person with community leaders introduced by the community health clinical authorities. Though informal interviews, leaders from the Ministry of Health, Ministry of Education, and Municipality Development Council (COMUDE) expressed concern regarding the high mortality rate associated with CKDu, the possible causes of CKDu, and need for timely detection, and accessible medical treatment, highlighting the high cost of kidney replacement therapy. These common themes are reflected in the quotes below:

“Kidney disease is a big problem in our community.” (Nurse, age 25)

“I have known neighbors that had chronic kidney disease, and they are not with us anymore.” (Principal of the school, age 38)

“I have known people that had to sell their land to have money for their hemodialysis treatment.” (Community leader, age 40)

“I know that there is a problem with kidney disease in our community, but I don’t know what to do.” (Medical assistant, age 32)

“I think that there are multiple causes of chronic kidney disease, to mention some are accessibility of good quality on water, hard work, bad food and fluid intake and unrestricted medication consumption”. (Medical director, age 48).

Once we had some insight regarding the perception of kidney health by the community and fluid intake information of people of the area, we identified influencers of charge who were also involved in community projects.

### Identification of participants

#### Participants

Community leaders from the Ministry of Health, Ministry of Education, and Municipality Development Council (COMUDE) were invited to be a part of the research team and the community engagement development program. Sixty-two out of the identified 145 members of the leadership group agreed to participate and represented the population of interest in engagement. All participants displayed a strong personal commitment to actively participate in the study. The quotes below reflect their level of engagement.

“It is sad to know that our municipality is on the top list of towns in Guatemala with a high prevalence of kidney issues, we are here to change that”. (Health program coordinator, age 38).

“We appreciate that you want to help our community, you can count on us.” (Community leader, age 36)

“We are grateful to your team for asking us to be a part of this change in our community.” (School supervisor, age 46).

### Design and planning

We scheduled weekly meetings with community leaders to develop proposals and structured research objectives and protocols to ensure a mutual benefit between the researchers and the community. During meetings, we created focus group discussions guided by a member of the research team and 10–12 community leaders to get ideas and insights in response to open-ended questions. The community leaders expressed their opinion about the methodology and the benefit of participation in the hydration project. We arranged study details, answered questions from the discussion and determined knowledge gaps. The focus group discussion meetings ranged between 45 to 60 min and the researcher moderated and obtained thoughtful responses by capturing ideas based on lifestyles, culture, attitudes, beliefs, perception and community environment. These ideas helped in establishing hydration study tools, such as the parent consent form, children assent form and fluid intake records during meetings. Community leaders actively participate in testing the instruments and provide feedback in the following meetings. These discussions helped with understanding the research goal, interpretation of findings and amplified the community voice. The research team made all their decisions by consensus with the community leaders.

### Knowledge integration on kidney health

We developed several educational activities to highlight the importance of kidney health and adequate fluid intake. The community leaders provided ideas, such as a community Kidney Walk and training for health professionals regarding screening of kidney health for the community. In March, during the World Kidney Day, the Kidney Walk occurred in La Democracia, the first one in rural Guatemala. Four hundred people participated in the walk, carrying educational banners about kidney health and messages encouraging kidney health through “the eight golden rules”. The eight golden rules promoted were to: be active, eat a healthy diet, check and control your blood sugar, check and control your blood pressure, have an appropriate fluid intake, avoid smoking, and avoid the regular intake of unprescribed anti-inflammatory/pain-killer pills.

As part of knowledge integration, 30 out of 45 health care personnel from the Community Clinic at La Democracia, participated in a course on prevention, early identification, and management of most common kidney diseases in the community. Six of the participants were part of the community leaders engaged in the program. We started the training by asking oral questions about their ideas on how to do an early detection and why was CKD important. A video was followed to answer introductory questions and to emphasize the importance of CKD. The training was held at the Community Clinic and lasted 1.5 h. We divided the participants into 3 groups with a nephrologist of the research team leading each group. The first group was composed by 14 nurses, the second group of 2 lab technicians and 8 health personnel. The topics discussed in both groups were: Risk factors associated with CKD, kidney health screening test and their interpretation (urine test, creatinine, blood pressure) with cases examples. The 3^rd^ group was composed of 6 general practitioners and the topics discussed were: generalities of CKD, a review of kidney health test and imaging, urinary tract infection, hypertension, glomerular diseases, and reasons for referral. At the end of the training a wrap-up session was held, the questions of the group were answered and the initial oral questions were again evaluated.

### Implementation and interpretation

With the community leaders as part of the research team, we started the planning for enrollment of participants in the hydration study and established specific tasks, including pilot testing of instruments, and conducted weekly updates before and during enrollment. By consensus community knowledge gaps were identified and adressed by the research team. During enrollment, leaders held open communication with the community, helped to disseminate information from the community by participating as data collectors or respondents to get input on the research project. This created a formal partnership and collaboration with community groups. Each community leaders recorded key experiences, thoughts, emotions and reactions in the field. Abstract concepts and generalizations which emanated from this process were documented as field notes and shared during updates. Extensive discussions were held to assist each community leader member to tackle any challenges they faced in the field of the enrollment process. Community leaders shared problem-solving, interpretation, and sharing results during research team updates. Insights are reflected in the quotes below:

“I tested the fluid intake questionnaire on my kid, and it was a bit of a challenge at the beginning. I suggest to do a training before enrollment and make sure with a pretest that the person that will fill the fluid intake questionnaire is capable to do it” (Community leader, age 40)

“I suggest that the fluid intake questionnaire have examples of different beverages sizes available, that way is easier to identified it on the questionnaire. (Teacher of Tierra Nueva, age 38).

“Regarding the type of fluid intake on the questionnaire, there are hot beverages in our community that people drink and are known as “atoles”. I suggest adding it on the category and naming a few examples on the list, that way we take into account an important fluid type of our community and don’t miss this information. (Community leader, age 36)

### Evaluation of the Community engagement program

Once the community engagement program was established over a 15-month period we evaluated the program by using a previously published, approved questionnaire [36]. At the end of the research project, we met with all community leaders and explained the structured questionnaire. Paper questionnaires were distributed, and details of qualitative and quantitative sections were explained to have a comprehensive understating of community engagement evaluation. Qualitative answers provided further understanding as to how well the research team adhered to engagement principles. Quantitative answers evaluated consistency of the responses as to how often the research team adhered. An explanation about answers scores were provided as higher scores corresponding to higher quality or frequency of adherence to the engagement principles.

### Questionnaire

The questionnaire evaluated five engagement principles that were translated into Spanish (additional file 1) using the World Health Organization translation guidelines [42] and was adapted for language clarity, cultural appropriateness, and conceptual understanding to:

- 1. Focus on local relevance and determinants of health of the community
- 2. Acknowledge of the community
- 3. Disseminate findings and knowledge gained to all partners.
- 4. Seek and use the input of community partners.
- 5. Involve a cyclical and iterative process in pursuit of objectives

Each engagement principle had four or five questions with “qualitative answers” and four to five questions with “quantitative answers” which comprised 46 questions in total. Half of the questions measure the quality (how well) research team's adherence to the five engagement principles. Each “qualitative answers” had the following response options on a 5-point Likert scale: Score 1 = Poor, 2 = Acceptable, 3 = Good, 4 = Very good, 5 = Excellent. The other half of the questions measure the quantity (how often) research team's adherence to the five engagement principles. Each “quantitative answers” had the following response options on a 5-point likert scale: Score 1 = Never, 2 = Rarely, 3 = Sometimes, 4 = Most of the time, 5 = Always.

### Analysis

The analysis of qualitative and quantitative responses for each engagement principle was described as median scores on each scale with and interquartile range. We assessed internal consistency for each set of the engagement items through Omega coefficient, and an item value set higher than 0.7 indicated agreement across answers.

## Results

The community leaders comprised six leaders of the Ministry of Health (a medical director, a medical assistant, a health administrator, a health program coordinator, a promotion officer and a nursing staff of the Health Community Center), 49 leaders of the Ministry of Education (four teachers, a principal and a supervisor per school) and seven leaders of COMUDE (a leader per each community of La Democracia: Tierra Nueva, La Bendición, La Promesa, La Unión, Cun Cun, Las Delicias, Parcelamiento El Pilar, Aldea El Pilar, El Milagro, Arenal). Seventy-five percent were female, with a mean age of 37 years (SD 9.8). All 62 community leaders completed the questionnaire after the development of the community engagement program (100% response rate to survey) Table 1.

**Table 1:** Characteristics of community leaders engaged (*N* = 62)

Characteristics	N	%
Gender		
Female	49	75.4
Male	16	24.6
Community leaders		
Ministry of Education (*N* = 49)		
Teachers	38	61.2
Principal	10	16.1
Supervisor	1	1.6
Municipal Development Council, COMUDE (*N* = 7)		
Tierra Nueva and La Bendición	1	1.6
La Promesa	1	1.6
La Unión	1	1.6
Cun Cun	1	1.6
Las Delicias	1	1.6
Parcelamiento El Pilar and Aldea El Pilar	1	1.6
El Milagro and Arenal	1	1.6
Ministry of Health (*N* = 6)		
Medical director	1	1.6
Medical assistant	1	1.6
Health administrator	1	1.6
Health program coordinator	1	1.6
Promotion officer	1	1.6
Nursing staff	1	1.6
	**Mean**	**SD**
Age (years)	37.1	9.8

### Qualitative results

For the full qualitative items across engagement principles, the median was five, corresponding to the maximum score of a 5-point Likert scale. The interquartile range for each item set was between four and five. Overall, participants felt that the academic partners adhere to five engagement principles between very good and excellent for qualitative items Table 2.

**Table 2:** Qualitative score and Internal consistency

Community leaders response *N* = 62 Engagement Principle	Median score (Q1, Q3)	Level of agreement (Omega coefficient)
**Engagement Principle 1: Focus on local relevance and social determinants of health**	**5 (4, 5)**	**0.88**
Focus on issues important to my community		
Focus on health problems that the community thinks are important		
Focus on the combined interaction of factors (i.e., personal, social, economic…) that influence health status		
Focus on cultural factors that influence health behaviors		
**Engagement Principle 2: Acknowledgment in the community**	**5 (4, 5)**	**0.83**
Show appreciation for community time and effort		
Highlight the community’s involvement		
Give credit to community members and others for work		
Value community perspectives		
**Engagement Principle 3: Dissemination findings and knowledge gained to all participants**	**5 (5, 5)**	**0.84**
Let community members know what is going on with the project		
Help community members with problems of their own		
Empower community members with knowledge gained from a joint activity		
Get findings and information to community members		
Help community members disseminate information using community publications		
**Engagement Principle 4: Seek and use the input of community partners**	**5 (5, 5)**	**0.85**
Ask community members for input		
Use the ideas and input of community members		
Change plans as a result of community input		
Involve community members in making key decisions		
Ask community members for help with specific tasks		
**Engagement Principle 5: Involve a cyclical and iterative process in pursuit of objectives**	**5 (5, 5)**	**0.88**
Share the results of how things turned out with the community		
Seek community input and help at multiple stages of the process		
Inform the community of what happened when their ideas were tried		
Plan for ongoing problem solving		
Involve the community in determining next steps		

### Quantitative results

Regarding all quantitative items across engagement principles, the median was five, corresponding to the maximum score of a 5-point Likert scale. The interquartile range for each item set was between four and five. Therefore, participants felt that the academic partners adhere to five engagement principles between most of the time and always for quantitative items Table 3.

**Table 3:** Quantitative score and Internal consistency

Community leaders response *N* = 62 Engagement Principle	Median score (Q1, Q3)	Level of agreement (Omega coefficient)
**Engagement Principle 1: Focus on local relevance and social determinants of health**	**5 (5, 5)**	**0.90**
Focus on issues important to my community		
Focus on health problems that the community thinks are important		
Focus on the combined interaction of factors (i.e., personal, social, economic…) that influence health status		
Focus on cultural factors that influence health behaviors		
**Engagement Principle 2: Acknowledgment in the community**	**5 (4, 5)**	**0.90**
Show appreciation for community time and effort		
Highlight the community’s involvement		
Give credit to community members and others for work		
Value community perspectives		
**Engagement Principle 3: Dissemination findings and knowledge gained to all participants**	**5 (4, 5)**	**0.88**
Let community members know what is going on with the project		
Help community members with problems of their own		
Empower community members with knowledge gained from a joint activity		
Get findings and information to community members		
Help community members disseminate information using community publications		
**Engagement Principle 4: Seek and use the input of community partners**	**5 (4, 5)**	**0.88**
Ask community members for input		
Use the ideas and input of community members		
Change plans as a result of community input		
Involve community members in making key decisions		
Ask community members for help with specific tasks		
**Engagement Principle 5: Involve a cyclical and iterative process in pursuit of objectives**	**5 (4, 5)**	**0.95**
Share the results of how things turned out with the community		
Seek community input and help at multiple stages of the process		
Inform the community of what happened when their ideas were tried		
Plan for ongoing problem solving		
Involve the community in determining next steps		

Every item set corresponding to an engagement principles evaluation had a Omega coefficient > 0.80, indicating a firm internal consistency for all question groups on both qualitative and quantitative scales.

## Discussion

Community engagement programs are a process to enhance success in performing research projects requiring community involvement and enrollment [8]. This study is the first community engagement program developed to support a kidney research study about hydration in an area in Guatemala with a high prevalence of CKDu. Our study demonstrates the process and the success of developing a community engagement program and the evaluation of how this research aligned with engagement principles in a quantitative measure.

When measuring the engagement, we obtained a 100% response rate to survey participants. The community engagement program evaluation described high standards of quality and frequency of engagement on each engagement principle. With a community engagement program, we were able to develop trust upon a strengthened relationship with the community based on mutual benefit, respect, honesty, and open communication.

Researchers faced challenges while recruiting participants because of mistrust of the research enterprises [3, 6, 8, 15, 43–47]. Engaging community members in our study helped to increase the understanding of CKDu, cultural factors, and health concerns that influence kidney health behaviors in the community. By including community members as part of the research group, instead of just research participants, there was a greater understanding and appreciation of the community involvement in the research process. Participants were able to adopt a new perspective on the importance of research and establish the role of the community by recommending social change efforts to address community concerns. This involvement resulted in a mutual benefit among all partners.

We learned that input from community members was a significant step in working towards the goal of engagement. The program also promoted an achievable goal with community members and promoted research that targeted outcomes that were relevant and useful outcomes to a community. This was possible through an intentional effort on listening, valuing, and demonstrating respect for community perspectives.

Although engaging a community in research requires planning and dedication, the program provided an opportunity to incorporate community input through educational activities [1, 6, 48]. The participants played a role as spokespeople and communicated an extensive and trusted body of information about kidney health. The engagement improved the ability to translate the message of the objective to real-world settings in the community by giving a simple message, easy to get, in a language that was understandable for the community.

Studies have shown successful results when using community engagement while doing research projects [49]. However, few of them have measured the level of engagement. The use of a structured questionnaire allowed us to determine how well and how often the research team objectively aligned with engagement principles. By using a qualitative and quantitative questionnaire, the results have shown that all item responses had a firm internal consistency representing agreement across replies.

## Conclusion

To our knowledge this is the first time publication assessing community engagement in the Guatemalan kidney research context. Assuring the engagement of the community in the kidney research projects provides sustainability of the efforts and facilitates the achievements of the goals. This community engagement program, provided valuable information about kidney health research and engagement. Community leaders and researchers became a team and develop a relationship in which commitment and empowerment facilitated participation in all aspects of the research process. This initiative could be a useful tool for researchers, especially in low-middle income countries, to start research in a community, achieve objectives in a viable form, and open opportunities to further studies.

## Supplementary Information

**Additional file 1.** Spanish version of community engagement assessment (Quantitative questionnaire). Spanish version of community engagement assessment (Qualitative questionnaire).

## Data Availability

All the data supporting this study are included within the manuscript and supplementary section. The dataset is available from the corresponding author.

## Abbreviations

CKD: Chronic Kidney Disease;
CKDu: Chronic Kidney Disease of Unknown cause;
COMUDE: Municipality Development Council
