# Supplementary material for: Community engagement in kidney research: Guatemalan experience
Source: BMC Nephrol. 2022 Aug 12;23:282. doi: 10.1186/s12882-022-02891-8 (PMC9373416; doi:10.1186/s12882-022-02891-8)

Spanish version of community engagement assessment (Qualitative questionnaire).


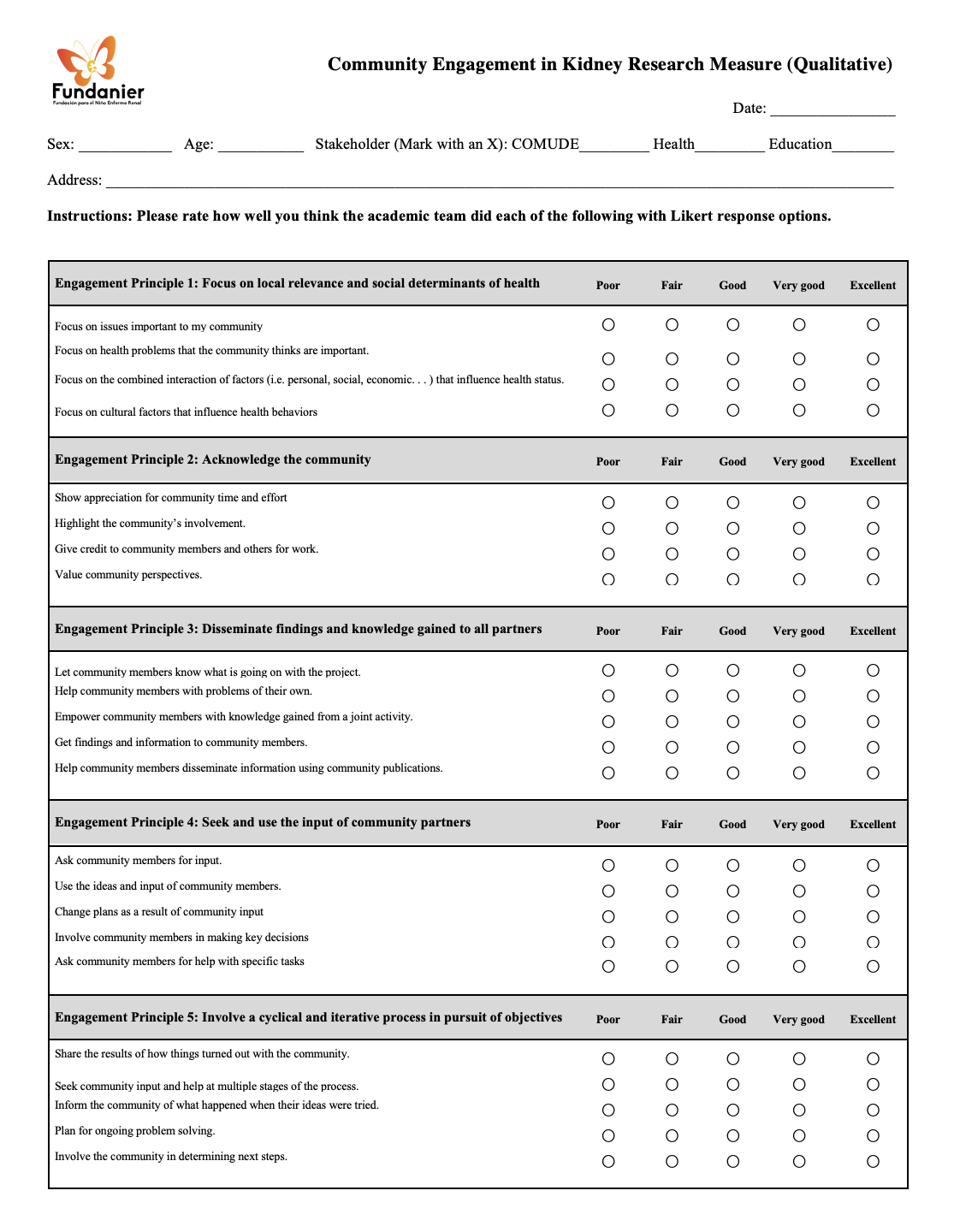


Spanish version of community engagement assessment (Quantitative questionnaire).


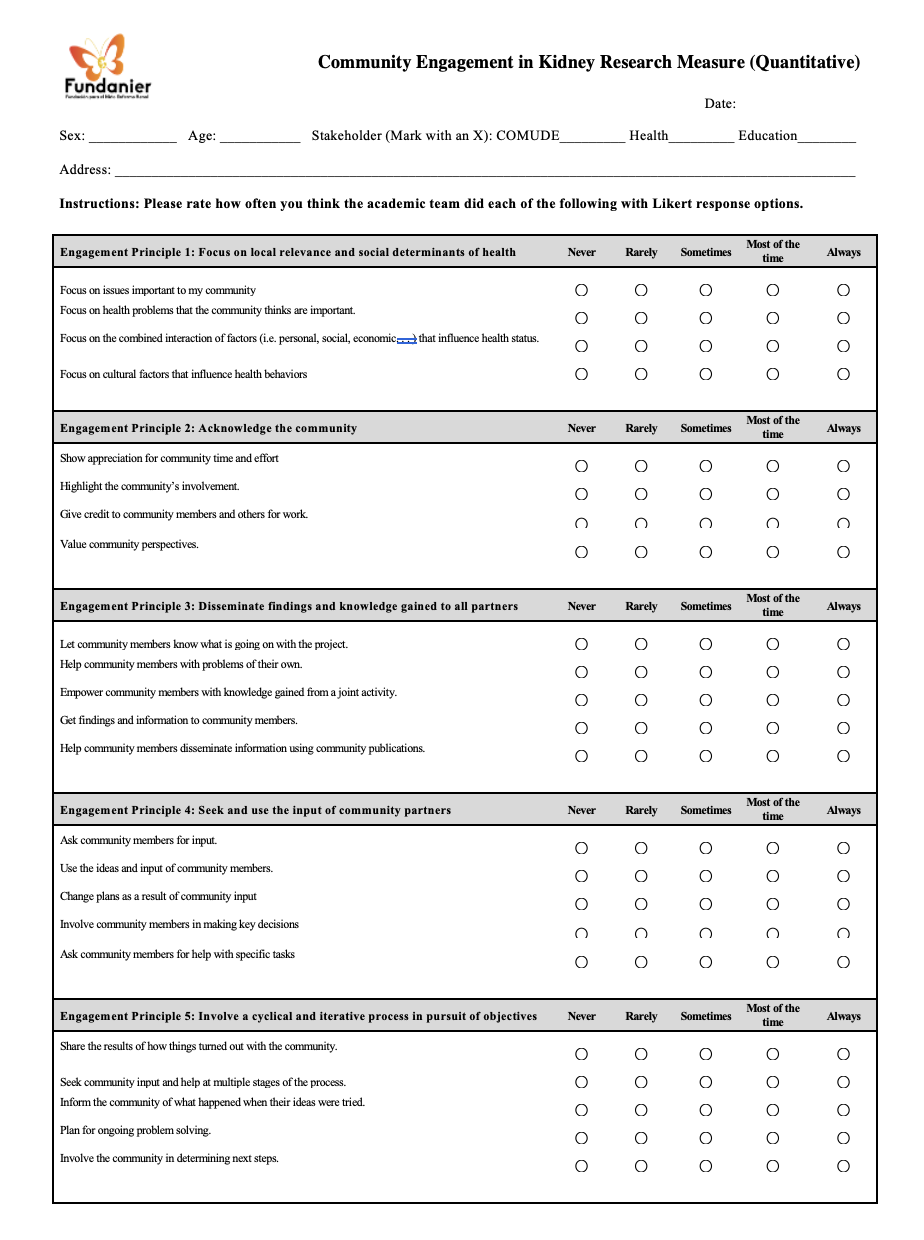

Supplement: Supplementary file 1 — Additional file 1. Spanish version of community engagement assessment (Quantitative questionnaire). Spanish version of community engagement assessment (Qualitative questionnaire). [file 12882_2022_2891_MOESM1_ESM.doc]
